# Retained Shrapnel from a Blast Injury as a Rare Cause of Secondary Osteoarthritis of the Hip Joint: A Case Report and Review of Literature

**DOI:** 10.1155/2019/7190781

**Published:** 2019-12-29

**Authors:** Oshan Basnayake, Ahamed Nihaj, Ranji Pitagampalage, Umesh Jayarajah, Yasith Mathangasinghe, Harsha Mendis

**Affiliations:** National Hospital of Sri Lanka, Colombo, Sri Lanka

## Abstract

**Background:**

Complications related to intra-articular retained shrapnel are rare and primarily depend on the anatomical location and the reaction with the surrounding tissue. Retained bodies causing severe osteoarthritis with bone destruction and limb shortening are extremely rare. We describe a rare occurrence of retained shrapnel, possibly iron nails causing a late presentation of grade 4 secondary osteoarthritis of the hip joint with destruction of the femoral head and limb shortening.

**Case Presentation:**

A 74-year-old otherwise healthy Sri Lankan male with a history of blast injury to the right hip 35 years ago presented with an isolated, right sided mild hip pain with a progressive limp for an 8-year duration. He had a true right limb shortening of 3.6 cm and limited range of motion. However, he had minimal functional disability. An X-ray of the pelvis and hip joints showed grade 4 osteoarthritis of the right hip joint with destruction of the right femoral head. There were three retained metallic nails (shrapnel) in the right hip joint of which two were intra-articular. Although he was offered a total hip arthroplasty, he opted for conservative management due to his minimal functional disability. Modified foot wear and simple analgesics were prescribed, and he had no worsening of symptoms at 6 months of follow up.

**Conclusion:**

Late presentation due to shrapnel-induced osteoarthritis with bone destruction and limb shortening is extremely rare. Initial assessment with radiographs is essential following blast injuries to exclude intra-articular or periarticular foreign bodies. Such foreign bodies should be removed to prevent the associated local and systemic complications.

## 1. Introduction

Shrapnel-induced injuries are commonly associated with gunshot and blast injuries, especially in war zones [1]. Complications related to retained fragments are rare and primarily depend on the anatomical location and the reaction with the surrounding tissue. Local and systemic complications due to retained intra-articular lead bodies have been described. However, retained bodies causing severe osteoarthritis with bone destruction and limb shortening are extremely rare. We describe a rare occurrence of retained shrapnel, possibly iron nails causing a late presentation of grade 4 secondary osteoarthritis of the hip joint with destruction of the femoral head and limb shortening.

## 2. Case Presentation

A 74-year-old otherwise healthy Sri Lankan male presented with an isolated, right sided mild hip pain with a progressive limp for an 8-year duration. His other joints were clinically unremarkable. He was able to carry out his activities of daily living and his job as a manual laborer without any need of analgesics. He gave a history of a bomb blast injury 35 years ago to his lower limbs mainly around the right hip. He received wound care and was discharged from the hospital as he had only mild pain during walking and no functional disability. His previous clinical records or radiographs were not available. He was averagely built with a body mass index (BMI) of 21.47 kg/m^2^. He had a true right limb shortening of 3.6 cm compared with the contralateral limb. Both active and passive ranges of motion of the right hip joint were restricted mainly with abduction and external rotation. The distal neurovascular examination was normal. His basic hematological and biochemical investigations were unremarkable.

**Figure 1:**
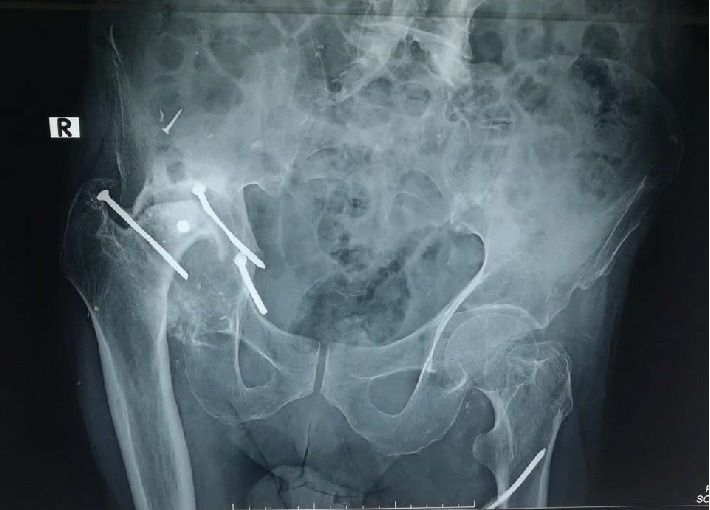
X-rays of the pelvis and both hip joint anteroposterior view showing grade 4 osteoarthritis of the right hip joint with destruction of the right femoral head due to retained shrapnel.

**Figure 2:**
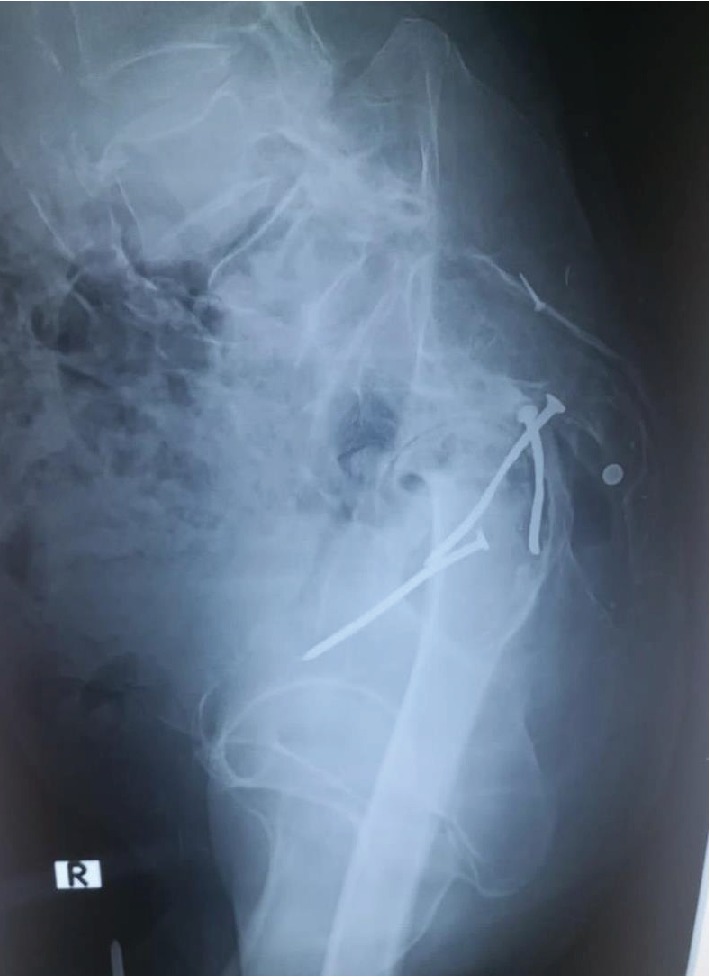
X-rays of pelvis and right hip joint lateral view showing grade 4 osteoarthritis of the right hip joint with destruction of the right femoral head due to retained shrapnel.

His X-rays of the hip joints revealed a destroyed femoral head with diffuse acetabular osteophyte formation. There were three retained metallic nails in the right hip joint of which two were intra-articular (Figures 1 and 2). The joint space was narrowed, and the presence of juxta-articular osteosclerosis was noted. The features were suggestive of secondary grade 4 osteoarthritis of the right hip joint. The contralateral hip joint and both knee joints were unremarkable. The patient was offered the option of a total hip arthroplasty. However, due to the mild clinical symptoms and minimal functional disability, the patient opted for conservative management. A modified foot wear was arranged to correct the length discrepancy, and simple analgesics were prescribed.

Follow-up after 6 months revealed good functional capacity without any worsening of symptoms.

## 3. Discussion and Conclusion

Blast- and gunshot-related injuries are common in countries in which war activities are prevalent. Such injuries are associated with a retained shrapnel or bullets. Long-term complications of retained shrapnel depend on the anatomical location and interaction with the tissues. A retained shrapnel deep within the muscle tissue rarely causes problems. However, complications due to shrapnel within the joint cavity due to either initial impact or subsequent migration have been reported [2–6]. The majority of the previously reported cases were related to retain lead bullets within the joint cavity or the periarticular region causing synovitis and systemic lead toxicity. We described a rare occurrence of retained shrapnel, possibly iron nails causing a late presentation of grade 4 secondary osteoarthritis with destruction of the femoral head and limb shortening.

Several theories have been postulated to explain the foreign body-induced arthropathy. Materials such as lead fragments were hypothesized to cause a local chemical reaction leading to synovitis and subsequent joint damage [7]. This argument was supported by the findings from the synovial biopsy examinations. Slavin et al. reported microscopic changes such as lead inclusion bodies in the nucleus, degranulated endoplasmic reticulum, mitochondrial precipitates in macrophages, and migration of osteoclasts [2]. Furthermore, lead containing extracellular complexes were also noted in an adjacent trabecular bone with incomplete osteocytic osteolysis, causing bone remodeling [2]. Several animal-based models after intra-articular lead implantation showed associated chondrocyte proliferation, changes of architecture of columnar epithelium, and cellular and stromal hyperplasia of synovium [8, 9]. In addition, unequal thickness of the articular cartilage and tide mark duplication were also observed. Sometimes other than this chronic osteoarthritis-type presentation, intra-articular foreign bodies can mimic the clinical picture of septic arthritis also [10]. Intra-articular lead poisoning causing gouty arthritis has also been reported [11].

In addition to the local effects, systemic lead toxicity by dissolution of material by the synovial fluid has also been described [3]. An inflamed synovium also facilitates the entry of dissolved lead into systemic circulation. Some of the systemic toxicity features following retained lead bullets include abdominal pain, renal impairment, encephalopathy, and anemia [4, 5]. Moreover, lead-induced severe hemolytic anemia has also been described [12]. Intriguingly, chelation and removal of the lead fragments from the joint caused the serum lead levels to normalize with clinical resolution of symptoms [3]. A few previous reports showed the drop in serum lead levels with time without surgical removal of lead particle [13, 14]. Rapid encapsulation of lead particles with fibrosis limits the exposure to synovial or other body fluids proposed as the mechanism for above phenomenon.

Other mechanisms for arthropathy may include primary injury-related articular cartilage damage or secondary migration of metal bodies into the joint cavity. This may subsequently cause progressive cartilage erosion and joint damage due to repeated mechanical insult during joint mobilization and weight bearing. The reported patient had been asymptomatic immediately after the injury. Therefore, the possibility of primary injury-related joint damage seemed unlikely. Furthermore, he became symptomatic after 26 years following the blast injury. Therefore, subsequent migration of the shrapnel in to the joint space may be the likely mechanism. However, the initial X-rays were not available for comparison. Similarly, in two patients, migration of iron fragment from the upper thigh to knee joint causing symptoms after 5 and 20 years following the initial injury was also reported [6, 15]. In both patients, the metal fragments were retrieved with the aid of arthroscopy. Arthroscopy has an additional advantage of examining the rest of the joint for other macroscopic changes related to joint damage.

Although iron is a relatively inert metal, local reaction with synovial fluid may cause joint destruction and subsequent osteoarthritis. Dissolution of lead fragments was explained by the pH value and hyaluronic acid of the synovial fluid [16]. Similarly, such mechanisms may cause dissolution of intra-articular iron fragments. Studies have been carried out to assess the impact of local iron concentration and iron chelation therapy in osteoarthritis and rheumatoid arthritis [17, 18]. Yazar et al. showed significantly higher concentration of synovial fluid iron and copper levels compared with age- and gender-matched healthy controls [17]. Intriguingly, similar finding was also noted in the histochemical examination of synovia in both osteoarthritis and rheumatoid arthritis [18]. Therefore, in addition to the mechanical damage caused by iron particles, chemical damage caused by high concentration of iron in the synovial fluid may be an important mechanism causing arthropathy.

However, further studies are needed to evaluate such local chemical effects of iron in the pathogenesis of osteoarthritis. Effects of the metals on the body depend on their biological activity, and an animal-based study showed that tungsten alloy material embedded in tissue can cause systemic inflammation with high-grade pleomorphic rhabdomyosarcomas which had high metastatic potential [19]. Tungsten alloy-based munitions are used as shrapnel in military practices recently.

The exact location of the foreign body can be determined by the use of computerized tomography scan (CT). Magnetic resonance imaging (MRI) modality cannot be used because magnetic field-induced migration of the foreign material can cause structural damage to local tissue [20].

The decision to proceed with the removal of foreign bodies depends on several factors. Initial wound debridement and removal of retained fragments may be needed depending on the initial wound size and degree of contamination [21]. Muscles and soft tissue-related fragments are usually encapsulated by a fibrous scar which limits the local reactions [22]. Retained fragments near neurovascular structures are at risk due to the possibility of local migration. Sometimes, leaving a known inert material may be advisable because removal can cause more harm than leaving it in situ. A case of delayed migration through vascular embolization of the shrapnel to the heart has also been reported [23]. Intra-articular shrapnel is usually advised to be removed due to the local and systematic complications.

In our patient, since the previous clinical records were not available, the reasons for not to proceed with the removal of shrapnel were not clear. The patient opted for conservative management due to the minimal symptoms and functional disability. This precluded the histological examination of the synovium which may have given interesting clues regarding the pathophysiological mechanisms.

## 4. Conclusion

Late presentation due to shrapnel induced osteoarthritis is rare. We described a rare occurrence of retained shrapnel, possibly iron nails causing a late presentation of grade 4 secondary osteoarthritis with destruction of the femoral head and limb shortening. Therefore, initial assessment with radiographs is essential following blast injuries to exclude intra-articular or periarticular foreign bodies. Such foreign bodies should be removed to prevent the associated local and systemic complications.

## Abbreviations

BMI: Body mass index
CT: Computerized tomography
MRI: Magnetic resonance imaging.

## Additional Points

*Availability of data and material.* All data generated or analyzed during this study are included in this published article.
